# Prevalence of Diabetes and Relationship with Socioeconomic Status in the Thai Population: National Health Examination Survey, 2004–2014

**DOI:** 10.1155/2018/1654530

**Published:** 2018-03-01

**Authors:** Wichai Aekplakorn, Suwat Chariyalertsak, Pattapong Kessomboon, Savitree Assanangkornchai, Surasak Taneepanichskul, Panwadee Putwatana

**Affiliations:** ^1^Department of Community Medicine, Faculty of Medicine, Ramathibodi Hospital, Rama VI Rd., Ratchathewi, Bangkok, Thailand; ^2^Faculty of Medicine, Chiang Mai University, Chiang Mai, Thailand; ^3^Faculty of Medicine, Khon Kaen University, Khon Kaen, Thailand; ^4^Epidemiology Unit, Faculty of Medicine, Prince of Songkla University, Songkhla, Thailand; ^5^College of Public Health Sciences, Chulalongkorn University, Bangkok, Thailand; ^6^Ramathibodi School of Nursing, Faculty of Medicine, Ramathibodi Hospital, Mahidol University, Bangkok, Thailand

## Abstract

**Objective:**

To determine the prevalence and trend of diabetes, related glycemic control, and influential socioeconomic (SES) factors in the Thai population aged ≥20 years during 2004–2014.

**Methods:**

Data from the Thai National Health Examination Survey 2004, 2009, and 2014 were used. Age-adjusted prevalence was calculated, and the associations of education levels with prevalence of diabetes and glycemic control were examined using logistic regression.

**Results:**

Age-adjusted prevalence of diabetes increased from 7.7% in 2004 to 7.8% in 2009 and 9.9% in 2014 (8.9% among men and 10.8% among women). Proportions of undiagnosed diabetes were slightly decreased but remained high in 2014 (51.2% for men and 41.3% for women). Diabetes prevalence was higher among those with primary education in both sexes; however, undiagnosed diabetes was higher among women with secondary and university educations. The percentages of those treated and controlled slightly improved among men (45.9%) but not among women (36.4%). Unmet glycemic control was also higher among women with secondary education levels and among men with university-level educations.

**Conclusions:**

Epidemic diabetes continues to grow in the Thai population, particularly in individuals with lower educational attainment. Measures to detect new cases and strengthen glycemic control should be scaled up.

## 1. Introduction

Diabetes is increasing worldwide, with escalating rates in the Western Pacific Region of Asia. The International Federation of Diabetes estimated that 415 million adults worldwide had diabetes in 2015 and this will rise to 642 million in 2040. Over 60% of people with diabetes worldwide live in Asia, with prevalence across countries ranging from 3% to 47.3% [1]. Diabetes is one of the major causes of cardiovascular diseases and microvascular disease complications. It is one of the nine global targets of the World Health Organization Global Action Plan for the Prevention and Control of Noncommunicable Diseases, with a target of zero increase by 2020. Monitoring of diabetes prevalence and management in terms of glycemic control among people with diabetes is an important component of health-care performance [2].

Thailand is among the countries in Asia with a high prevalence of diabetes [1]. More than 200,000 deaths annually among the Thai population are owing to chronic noncommunicable diseases, and about 30,000 deaths are owing to diabetes, a leading cause of death in Thailand [3]. Previous Thai National Health Examination Surveys (NHES) have shown that the prevalence of diabetes in individuals aged 20 years and over increased from 7.1% in 2004 to 7.5% in 2009 [4, 5]. The proportion of unawareness of diabetes declined from 66% in 2004 to 33% in 2009 whereas the proportions of treatment and control for all diabetes increased from 15% in 2004 to 31% in 2009. Recent studies in several countries, such as China and Korea, have reported increasing trends of diabetes whereas prevalence has remained relatively stable in countries like the United States and Japan [6]. There is evidence that diabetes prevalence is differentially distributed by socioeconomic status (SES). The association between SES and prevalence of diabetes varies according to the level of national economic development. In developing countries, educational attainment as a proxy for SES has been positively associated with diabetes whereas this relationship was the opposite in developed countries [7–9]. Furthermore, studies have demonstrated that this relationship is modified by sex [7, 10]. A previous study in the Thai population showed that obesity was higher among women with primary-level education whereas the prevalence was higher among men with secondary education levels and above [11]. It is of interest to determine the distribution of diabetes variability by educational level and sex. Periodic survey data on the prevalence and management of diabetes provide information that can be used to improve health-care performance. The fifth Thai NHES (NHES V) was conducted in 2014. The present study aimed to determine the prevalence and management of diabetes among Thai adults in 2014 and trends between 2004 and 2014, using data from NHES III, IV, and V.

## 2. Research Design and Methods

### 2.1. Sampling Methods

The NHES is a cross-sectional survey of a representative noninstitutionalized Thai population and is carried out every 5 years. The survey applies multistage sampling methods. In brief, the four-stage sampling method is as follows: (1) five provinces in each of the four regions of Thailand, and Bangkok, are selected; (2) two to three districts are randomly selected from each province; (3) 24 enumeration areas (EAs) are randomly selected from each province, with 12 EAs in urban and 12 EAs in rural areas, for a total of 540 EAs; (4) individuals of both sexes from each age group (15–29, 30–44, 45–59, 60–69, 70–79, and 80 years or more) are randomly selected from each EA. For the NHES V in 2014, the final sample size for participants aged 20 years and above was 22,095 participants. Finally, a total of 18,066 adults with available blood samples were included (81.8%). Sampling methods in 2004 and 2009 were similar to that in 2014 survey.

### 2.2. Data Collection

The survey was approved by the Ethical Review Committee for Research in Human Subjects, Faculty of Medicine, Ramathibodi Hospital, Mahidol University. Data were collected in face to-face interviews conducted by research nurses using a questionnaire at community centers, such as district hospitals, schools, or temples. Blood pressure was measured using a standard automatic blood pressure monitor (Omron model HEM-7117, Omron HealthCare Co. Ltd., Kyoto, Japan). Each participant was seated for at least 5 minutes before the first of three serial blood pressure measurements at 1-minute intervals. For each participant, the average of the second and third reading was used. Weight and height were measured using standard procedures. Body mass index was calculated according weight (kg)/height^2^ (m^2^).

Blood samples were obtained from participants who were asked to fast overnight for 8 hours. The samples were transferred for testing to provincial hospitals for determination of fasting plasma glucose (FPG) using an enzymatic hexokinase method. All the provincial laboratories were standardized to the central laboratory at the Department of Medical Service, Ministry of Public Health. Low-density lipoprotein cholesterol (LDL-C) was measured by enzymatic methods at the central laboratory of Ramathibodi Hospital.

### 2.3. Definitions of Diabetes

Diabetes was defined as individuals with a history of previous diagnosis by a physician plus having taken antihypoglycemic medication within the past 2 weeks. Diabetes was also considered in individuals with FPG levels of 126 mg/dL or greater at the time of data collection. Undiagnosed diabetes was defined as participants whose FPG level was 126 mg/dL or greater and reported that they had never been diagnosed with diabetes. Impaired fasting plasma glucose (IFPG) was considered in participants who had never been diagnosed with diabetes but with FPG levels between 100 mg/dL and 125.9 mg/dL.

### 2.4. Diabetes Awareness, Treatment, and Control

Diabetes that was considered treated and controlled referred to participants who received treatment with glucose-lowering medication and with FPG < 130 mg/dL. The percentage of treated and controlled was represented by two indicators with a common numerator, that is, the number of participants with diabetes and FPG < 130 mg/dL. In the first indicator, the numerator was divided by the number of participants with all diabetes (combined known and unknown diabetes); in the second indicator, the numerator was divided only by the number of participants who were aware of and treated for diabetes.

### 2.5. Statistical Analysis

The analysis took into account the complex survey design, as all of the estimates were weighted according to the inverse of probability of being sampled based on the 2014 registered Thai population. The prevalence of diabetes, undiagnosed diabetes, and IFPG was calculated. The prevalence was calculated for overall population stratified by age, sex, area of residence (urban/rural), and educational level (no formal education or primary, secondary, and university levels). We calculated age-standardized prevalence of all diabetes for 2004, and 2009 to the 2014 population. To examine trends of prevalence over time, the prevalence was regressed on the midpoint of each survey period as a continuous variable by logistic regression. Multiple logistic regression was used to examine the associations between education attainment and prevalence of diabetes, undiagnosed diabetes, and poor glycemic control, adjusted for age and area of residence and using separate models for men and women.

All statistical analyses were performed using Stata version 13.0 software (StataCorp., College Station, TX, USA). The significance level was two-sided, and *P* values were set at <0.05.

## 3. Results

Table 1 shows that the overall prevalence of diabetes in Thai adults aged ≥20 years was 9.9% (95% CI: 9.4%, 10.4%), with higher prevalence among women than among men: 10.8% (95% CI: 10.2%, 11.4%) and 8.9% (95% CI: 8.3%, 9.5%), respectively. Prevalence of diabetes was higher in rural areas than in urban areas for women (*P* = 0.03) but not for men. The prevalence of undiagnosed and diagnosed diabetes was 4.1% (95% CI: 3.7%, 4.5%) and 5.8% (95% CI: 5.5%, 6.1%), respectively. Diagnosed diabetes was higher in women (6.8% [95% CI: 6.4%, 7.2%]) than in men (4.6% [95% CI: 4.3%, 5.0%], *P* < 0.05). Overall, age-standardized prevalence for IFPG was 15.4% (95% CI: 14.7%, 16.2%), and the prevalence was slightly higher among men with 16.3% (95% CI: 15.4%, 17.3%) than among women with 14.6% (95% CI: 13.7%, 15.5%), but with no significant difference. The age-specific prevalence of diabetes increased with age and peaked at 60–69 years old, plateauing thereafter in men but declining slightly among women aged 70 years and older.

For both sexes, the prevalence of IFPG, undiagnosed diabetes, and diagnosed diabetes was not significantly different between urban and rural areas. The age-standardized prevalence of diabetes was significantly lower among participants with higher educational levels; this was particularly pronounced for women.

Table 2 depicts the age-standardized prevalence of diabetes in 2004, 2009, and 2014. Increasing trends of diabetes prevalence were observed among both men and women (all *P* values for trend < 0.001). The age-standardized prevalence of diabetes significantly increased in those aged 60 years and over, from 12.8% in 2004 to 15.3% in 2014 for men and from 16.3% to 20.3% for women (all *P* values for trend < 0.001). For age groups 20–39 years and 40–59 years, the age-standardized prevalence remained relatively stable during 2004, 2009, and 2014.

Diabetes prevalence was significantly increased over time among women living in both urban and rural areas; only men living in rural areas had increased prevalence. When stratified by educational level, diabetes increased among men with primary and secondary education levels. For women, diabetes increased only among those with no formal education or primary education levels (all *P* values < 0.001).

Between 2004 and 2014, there were no significant changes in the percentages of hypertension, dyslipidemia (LDL-C ≥ 100 mg/dL), and overweight and obesity (BMI 25–29.9 kg/m^2^ and BMI ≥ 30 kg/m^2^) among people with diabetes. In 2014, 46.4% of men and 48.1% of women with diabetes had hypertension. Seventy percent of people with diabetes have LDL-C > 100 mg/dL. The percentages for LDL-C ≥ 100 mg/dL declined slightly in the most recent survey; however, these did not change significantly. About half of the people with diabetes have BMI ≥ 25 kg/m^2^, with 13.5% of men and 20% of women having BMI ≥ 30 kg/m^2^. However, the percentage of individuals who had diabetes with hypertension and controlled BP under 140/90 mmHg increased significantly, from 9.7% in 2004 to 33.6% in 2014 for men; among women, the corresponding percentages were 16.5% and 45.3%.

Table 3 depicts the age-standardized percentages of undiagnosed diabetes, treated, and controlled over time. The proportion of undiagnosed diabetes among men declined significantly from 65.2% in 2004 to 51.2% in 2014; this did not change significantly among women, with 48.5% in 2004 and 41.3% in 2014. Only a small percentage of diabetes cases were untreated; however, among all diabetes, about one fifth had controlled FPG with levels under 130 mg/dL. In 2014, the percentages of glycemic control, with FPG ≤ 130 mg/dL among those treated, were 45.9% for men and 36.4% for women. The percentages of treatment and control improved significantly among men but not among women.

Table 4 shows the adjusted ORs for factors related to diabetes prevalence, undiagnosed diabetes, and inadequate glycemic control among those treated. The adjusted ORs of diabetes prevalence were higher among participants with primary education levels compared with those with university education levels: 1.3 (95% CI: 1.1, 1.5) in men and 2.2 (95% CI: 1.4, 3.3) in women. However, compared with participants who had a primary-level education, undiagnosed diabetes was higher in women with secondary- and university-level educations: 1.7 (95% CI: 1.3, 2.2) and 3.8 (95% CI: 2.7, 5.3), respectively. The odds of undiagnosed diabetes were higher in younger age group in both sexes and among women in rural areas compared to urban areas. The ORs of unmet glycemic control were also higher among women with secondary and men with university education levels.

## 4. Discussion

The present study is the first to report the national prevalence and trends of diabetes in the Thai population over 10 years. The diabetes prevalence in Thailand has been increasing dramatically during the past decade, from 7.0% in 2004 to 9.7% in 2014. Our results show a higher diabetes prevalence among people of both sexes with lower educational attainment, with a more pronounced pattern among women. There remains a gap in access to diagnosis and quality of care; the percentages of undiagnosed diabetes and meeting the glycemic control target were 40% and 43%, respectively. Undiagnosed diabetes was highest for the youngest age group in this study. Poor glycemic control was high in the middle age groups for both sexes and for men with university-level and women with secondary-level educations.

The increased prevalence of diabetes is likely to be related to multiple factors including environmental one that affect lifestyle, especially unhealthy dietary patterns and decreased physical activity levels. The Thai diet is characterized by high intake of carbohydrates, sweets, and fat. White rice, a staple in Thailand, as well as sticky rice with a high glycemic index, has been found to be associated with increased risk of diabetes [12]. Our previous study showed that low physical activity together with a high carbohydrate intake pattern increased the risk of metabolic syndrome [13]. Urbanization and dietary patterns are likely to be the main contributing factors to the increase in diabetes prevalence [14]. The rapid rise in diabetes prevalence concurs with findings in other Asian countries, such as Malaysia, Singapore, China, South Korea, and Taiwan [6]. In Malaysia, the prevalence of diabetes increased from 11.6% in 2006 to 15.2% in 2011 and 22.9% in 2013 [15]. Diabetes prevalence in Singapore in 2010 was 11.3% [16]. However, these studies used the oral glucose tolerance test to define diabetes; therefore, comparisons of prevalence must be made with caution. The diabetes prevalence in the Thai population is relatively similar to that in South Korea, where prevalence was 10.1% in 2010–2012, based on FPG [8], and that in Japan, which has remained relatively stable at approximately 10% [6]. However, the diabetes prevalence in the present study was much higher than that in Vietnam and Cambodia, which is likely owing to differences in detection methods, age structure, the degree of urbanization, and lifestyle.

The higher proportion of undiagnosed diabetes in the latest survey was surprising. Given the national program to promote identification of people with hyperglycemia, obesity, and high blood pressure, the percentage of people with access to screening tests was disappointedly low, indicating that a number of people remain outside the screening program. Our analysis identified that participants with the lowest access to testing were those in the youngest age group. Compared with other countries, the proportions of undiagnosed diabetes are slightly lower in the United States (36.4%) [17] and Korea (32%) [18]. The proportion of treated patients who had adequate glycemic control is similar to that in China, with 39.7% [19]. The high percentage of unawareness of diabetes in 2014 suggests that there is room for improvement of screening programs and diabetes care.

Despite the increased prevalence, the percentages of obesity, hypertension, and dyslipidemia were relatively stable, and the percentage of controlled blood pressure was significantly improved. In the past 10 years, there has been a move to improve the quality of care through disease management and standard of care practices. The Thai Health Security Office, in association with the Ministry of Public Health, Thai Association of Diabetes, and the Association of Endocrinology, established an updated national clinical practice guideline to enhance the quality of care for diabetes. Support systems that include training of provincial health system managers and hospital case managers have been established; however, the results of these initiatives have not been evaluated. Our study results might play a role as a monitoring tool to evaluate clinical improvement [20].

The present study demonstrates differential changes in diabetes prevalence by area of residence, with higher rates of increase for both sexes in rural areas than in urban ones, in the two most recent surveys. The urban–rural gap was already narrow in the previous survey; the difference in urban–rural change for the most recent survey was such that diabetes prevalence was higher among women in rural areas than in urban ones. An important factor for the increase in diabetes is obesity, which was increasing at a higher rate in the rural areas than in the urban areas attributable to the change in lifestyle of rural residents to high-calorie food intake and less physically active [11]. This coincides with findings in the other developing countries of Asia where the prevalence is rising rapidly [1, 21] such as India, Nepal, Sri Lanka, Solomon Islands, and China. Obesity plays a substantial role in this increase of diabetes, with overweight and obesity showing an increasing trend in 2009 and 2014 [11]. Recently, a study among 10 Asian countries showed that Thai women were ranked second to Malaysia for the highest average BMI [22]. Other factors that might contribute to this rising prevalence might be related to epigenetic factors, which require further investigation.

Education is a proxy for SES and health literacy, and it has been found that low educational level is associated with high prevalence of diabetes in Canadian and Korean populations [7, 8]. In our study, the prevalence of diabetes was higher in participants with lower educational attainment in both men and women, but the magnitude of difference was greater among women. A study in the United States showed that diabetes prevalence has increased from 1988 to 2012 at all educational levels, with higher prevalence among people with lower educational levels [17]; however, that study did not mention whether the pattern differed by sex. In Korea, individuals with the lowest incomes had a 1.3 times greater risk of diabetes compared with people of high income levels. In addition, people with low educational attainment had higher likelihood of developing diabetes compared with those with higher educational levels, and the gradient seemed to be greater among women [8]. The positive association between socioeconomic status and diabetes was also observed in a Thai cohort study [23]. People with higher educational levels might have better access to health information and be more concerned with their health, leading to a healthier diet and lifestyle and less obesity [11]. However, some findings require further investigation, such as the higher proportion of undiagnosed diabetes among women with higher education, which implies that these women are less likely to be screened owing to certain reasons or barriers. In addition, the higher rate of inadequate glycemic control among men with higher education warrants further research.

The strengths of the present study include the use of nationally representative data from a series of national surveys, to determine the prevalence and trends of diabetes in the Thai population. A limitation of the study is the use of FPG to define diabetes in the present study, which might underestimate the number with impaired glucose tolerance but without abnormal FPG levels. The diabetes prevalence is likely to be underestimated by the use of FPG; we would expect that more cases will be found by measuring glucose tolerance or by combining this with glycated hemoglobin testing.

In conclusion, we found a continuing rise in diabetes among the population in Thailand, particularly among individuals with low educational levels. Improvement in the identification of people with elevated risk and implementation of effective lifestyle modification programs are needed to lower diabetes incidence. Gaps in access to diagnosis were highest in the youngest age group, and the quality of glycemic control must be improved across educational levels, particularly for women with secondary-level educations. Barriers to accessing screening programs and factors related to inadequate glycemic control should be investigated.

## Figures and Tables

**Table 1:** Prevalence of diabetes in Thai participants aged ≥20 years (NHES V, 2014).

	% (95% CI)					
	*n*	IFPG	Undiagnosed diabetes	Diagnosed diabetes	All diabetes	*P* value
*All*	18,066	15.4 (14.7, 16.2)	4.1 (3.7, 4.5)	5.8 (5.5, 6.1)	9.9 (9.4, 10.4)	
*Men*	7546	16.3 (15.4, 17.3)	4.3 (3.9, 4.7)	4.6 (4.3, 5.0)	8.9 (8.3, 9.5)	
*Age (y)*						
20–29	559	6.2 (5, 7.8)	1.5 (1.0, 2.3)	0.1 (0.03, 0.2)	1.6 (1.1, 2.3)	<0.01
30–39	801	15.6 (13.9, 17.6)	3.0 (2.3, 3.9)	0.5 (0.3, 0.8)	3.5 (2.8, 4.4)	
40–49	1383	16.2 (14.9, 17.7)	4.8 (4.2, 5.6)	3.2 (2.7, 3.8)	8.1 (7.2, 9.1)	
50–59	1566	20.5 (18.9, 22.2)	5.8 (5.2, 6.6)	7.7 (6.9, 8.5)	13.5 (12.4, 14.7)	
60–69	1900	20.8 (19.3, 22.3)	5.6 (4.9, 6.4)	10.5 (9.5, 11.5)	16.1 (14.9, 17.4)	
70+	1337	20.9 (19.3, 22.7)	4.7 (4.1, 5.5)	9.5 (8.5, 10.6)	14.2 (13.0, 15.6)	
^∗^ *Area of residence*						
Urban	3608	14.7 (13.8, 15.7)	3.9 (3.6, 4.3)	4.5 (4.1, 4.9)	8.4 (7.9, 9.0)	0.93
Rural	3938	16.4 (15.3, 17.7)	4.1 (3.5, 4.8)	3.9 (3.5, 4.3)	8.1 (7.2, 9.0)	
^∗^ *Educational level*						
≤Primary	4731	15.8 (14.5, 17.1)	4.7 (4.2, 5.2)	4.1 (3.7, 4.5)	8.8 (8.1, 9.4)	<0.01
Secondary	2100	14.7 (13.5, 16.0)	3.9 (3.4, 4.5)	4.6 (4.2, 5.1)	8.6 (7.8, 9.3)	
University	715	18.6 (17.0, 20.4)	3.3 (2.7, 4.2)	4.1 (3.4, 4.9)	7.4 (6.5, 8.5)	
*Women*	10,520	14.6 (13.7, 15.5)	3.9 (3.5 4.4)	6.8 (6.4, 7.2)	10.8 (10.2, 11.4)	
*Age (y)*						
20–29	732	8.9 (7.0, 11.1)	2.9 (1.9, 4.2)	0.3 (0.1, 0.5)	3.1 (2.2, 4.5)	<0.01
30–39	1230	10.8 (9.6, 12.2)	3.1 (2.3, 4.2)	1.3 (1.0, 1.7)	4.4 (3.6, 5.5)	
40–49	2104	14.2 (13.1, 15.4)	4.2 (3.5, 5.0)	4.0 (3.5, 4.6)	8.2 (7.4, 9.1)	
50–59	2356	17.4 (16.2, 18.6)	4.4 (3.9, 4.9)	9.7 (8.8, 10.6)	14.1 (13.1, 15.2)	
60–69	2453	19.0 (17.6, 20.4)	5.2 (4.5, 6.1)	16.6 (15.5, 17.8)	21.9 (20.5, 23.3)	
70+	1645	18.9 (17.4, 20.6)	3.9 (3.3, 4.7)	14.5 (13.2, 15.8)	18.4 (16.8, 20.1)	
^∗^ *Area of residence*						
Urban	5956	13.9 (12.8, 15.0)	3.5 (3.0, 4.1)	6.6 (6.2, 7.0)	10.1 (9.4, 10.8)	0.03
Rural	4564	14.5 (13.5, 15.7)	4.2 (3.5, 4.9)	6.3 (5.9, 6.8)	10.5 (9.7, 11.4)	
^∗^ *Educational level*						
≤Primary	7138	15.2 (14.1, 16.6)	3.8 (3.4, 4.2)	7.4 (6.9, 7.9)	11.1 (10.5, 11.7)	<0.001
Secondary	2368	16.1 (14.5, 17.7)	3.0 (2.5, 3.5)	4.5 (3.8, 5.2)	7.4 (6.6, 8.2)	
University	1014	10.5 (9.1, 12.2)	2.9 (1.7, 5.0)	2.3 (1.7, 3.1)	5.3 (3.6, 7.7)	

CI: confidence interval; IFPG: impaired fasting plasma glucose.

^∗^Age-standardized to the 2014 Thai population.

**Table 2:** Age-standardized prevalence of diabetes and prevalence by area of residence and educational level in the Thai population aged ≥20 years in 2004, 2009, and 2014.

	Men: % (95% CI)				Women: % (95% CI)			
	2004 (*n* = 18,963)	2009 (*n* = 8803)	2014 (*n* = 7546)	*P* value	2004 (*n* = 21,149)	2009 (*n* = 9826)	2014 (*n* = 10,520)	*P* value
Number with diabetes	2186	895	896		2822	1177	1440	
Overall prevalence	7.1 (6.6, 7.6)	6.8 (6.1, 7.6)	8.9 (8.3, 9.5)	<0.01	8.3 (7.9, 8.9)	8.7 (8.0, 9.5)	10.8 (10.2, 11.4)	<0.01
*Age (y)*								
20–39	3.3 (2.7, 4.1)	2.2 (1.5, 3.1)	2.5 (1.7, 3.7)	0.16	3.0 (2.4, 3.7)	1.6 (1.0, 2.5)	3.8 (2.9, 5.1)	0.23
40–59	9.7 (8.8, 10.7)	7.5 (6.2, 8.9)	10.9 (9.6, 12.5)	0.06	10.9 (9.9, 11.9)	9.7 (8.4, 11.2)	11.3 (10.0, 12.7)	0.47
≥60	12.8 (12.0, 13.6)	13.8 (12.3, 15.5)	15.3 (13.8, 17.1)	<0.01	16.3 (15.5, 17.2)	17.6 (16.3, 19.0)	20.3 (18.6, 22.0)	<0.001
*Area of residence*								
Urban	9.5 (8.9, 10.2)	9.3 (8.0, 10.7)	8.4 (7.9, 9.0)	0.88	9.1 (8.6, 9.7)	10.3 (9.3, 11.3)	10.1 (9.4, 10.8)	0.02
Rural	6.3 (5.7, 7.0)	5.7 (4.9, 6.7)	8.1 (7.2, 9.0)	<0.001	8.1 (7.5, 8.7)	8.0 (7.0, 9.1)	10.5 (9.7, 11.4)	<0.001
*Educational level*								
≤Primary	7.6 (6.9, 8.3)	7.3 (6.3, 8.4)	8.8 (8.1, 9.4)	<0.001	9.4 (8.8, 10.1)	10.1 (9.1, 11.2)	11.1 (10.5, 11.7)	<0.001
Secondary	7.3 (6.4, 8.3)	7.0 (5.9, 8.4)	8.6 (7.8, 9.3)	0.27	6.8 (5.7, 8.2)	6.3 (5.1, 7.9)	7.4 (6.6, 8.2)	0.06
University	8.8 (7.3, 10.6)	6.1 (3.8, 9.4)	7.4 (6.5, 8.5)	0.86	5.6 (4.1, 7.6)	5.8 (3.4, 9.7)	5.3 (3.6, 7.7)	0.73
*Comorbidity*								
Hypertension	45 (41.2, 49.1)	43.0 (36.8, 49.5)	46.4 (41.1, 51.8)	0.69	47.3 (43.7, 51.1)	48.3 (42.6, 54.1)	48.1 (43.6, 52.7)	0.10
BP controlled < 140/90 mmHg	9.7 (7.4, 12.6)	24.6 (16.8, 34.5)	33.6 (26.6, 41.5)	<0.01	16.5 (13.3, 29.3)	34.4 (27.0, 42.7)	45.3 (39.0, 51.6)	<0.01
High LDL-C ≥ 100 mg/dL	NA	70.5 (63.5, 76.7)	68.8 (63.4, 73.7)	0.67	NA	77.0 (71.7, 81.5)	71.3 (66.5, 75.7)	0.11
BMI ≥ 25–29.99 kg/m^2^	31.6 (28.2, 35.2)	34.6 (28.5, 41.2)	35.0 (30.1, 40.3)	0.51	35.5 (32.1, 39.1)	41.6 (36.0, 47.4)	38.1 (33.8, 42.6)	0.27
BMI ≥ 30 kg/m^2^	12.1 (9.7, 14.9)	14.7 (10.5, 20.1)	13.5 (10.2, 17.5)		20.0 (17.1, 23.3)	21.4 (17.0, 26.6)	19.8 (16.5, 23.5)	

CI: confidence interval; BP: blood pressure; LDL-C: low-density lipoprotein cholesterol; BMI: body mass index.

**Table 3:** Age-standardized percentage of awareness and treatment of diabetes among Thai adults aged ≥20 years with diabetes, according to sex in 2004, 2009, and 2014.

	Men: % (95% CI)			Women: % (95% CI)		
	2004	2009	2014	2004	2009	2014
Undiagnosed	65.2 (61.2, 69.0)	46.1 (39.4, 53.0)	51.2 (45.9, 56.6)	48.5 (44.6, 52.5)	23.3 (18.7, 28.6)	41.3 (36.6, 46.1)
Diagnosed but not treated	2.0 (1.2, 3.2)	5.4 (2.6, 11.1)	4.2 (2.4, 7.4)	1.6 (1.0, 2.6)	1.9 (1.0, 3.6)	1.7 (0.9, 2.9)
Treated but not controlled	23.7 (20.4, 27.4)	29.7 (24.1, 36.0)	23.9 (19.9, 28.3)	32.0 (28.5, 35.6)	40.1 (34.6, 46.0)	35.7 (31.5, 40.2)
Treated and controlled, among all diabetes	9.1 (7.3, 11.3)	18.8 (14.8, 23.4)	20.7 (16.9, 25.0)	17.9 (15.2, 21.0)	34.7 (29.4, 40.3)	21.3 (18.1, 25.0)
Treated and controlled, among those treated	26.3 (20.7, 32.8)	36.9 (29.0, 45.6)	45.9 (38.5, 53.5)	35.5 (30.5, 40.9)	46.1 (39.5, 52.9)	36.4 (30.9, 42.3)

Undiagnosed diabetes was defined as participants whose FPG level was 126 mg/dL or greater and reported that they had never been diagnosed with diabetes. Diabetes that was considered treated and controlled referred to participants who received treatment with glucose-lowering medication and FPG < 130 mg/dL. The percentage of treated and controlled among all diabetes was the number of participants with diabetes and FPG < 130 mg/dL divided by the number of all participants with diabetes (combined known and unknown diabetes) and for those treated and controlled among treated was the number of participants with diabetes and FPG < 130 mg/dL divided by the number of participants who were aware of and treated for diabetes.

**Table 4:** Adjusted odds ratios for prevalence of diabetes, undiagnosed, and uncontrolled among those treated (NHES V, 2014).

	Men: % (95% CI)			Women: % (95% CI)		
	Diabetes	Undiagnosed	Uncontrolled	Diabetes	Undiagnosed	Uncontrolled
*Age (y)*						
20–39	1	38.3 (18.3, 80.4)	1.0 (0.2, 3.2)	1	9.8 (6.3, 15.4)	1.3 (0.6, 2.9)
40–59	6.3 (4.6, 8.6)	2.1 (1.7, 2.5)	1.4 (1.0, 1.9)	3.0 (2.4, 3.7)	2.1 (1.7, 2.6)	1.9 (1.6, 2.3)
≥60	9.8 (7.1, 13.6)	1	1	5.7 (4.4, 7.2)	1	1
*Residence*						
Urban	1	1	1	1	1	1
Rural	0.9 (0.7, 1.0)	0.9 (0.7, 1.1)	0.7 (0.5, 0.9)	0.9 (0.8, 1.0)	1.3 (1.1, 1.5)	0.8 (0.6, 1.1)
*Educational level*						
≤Primary	1.3 (1.1, 1.5)	1	1	2.2 (1.4, 3.3)	1	1
Secondary	1.1 (0.9, 1.3)	1.0 (0.8, 1.2)	1.0 (0.7, 1.3)	1.2 (0.8, 1.9)	1.7 (1.3, 2.2)	1.9 (1.4, 2.7)
University	1	1.0 (0.7, 1.4)	3.7 (1.9, 7.3)	1	3.8 (2.7, 5.3)	0.2 (0.1, 0.5)

Variables adjusted in the model included age, area of residence (urban/rural), and educational level.
